# Integration of light and hormone signaling pathways in the regulation of plant shade avoidance syndrome

**DOI:** 10.1007/s42994-021-00038-1

**Published:** 2021-04-26

**Authors:** Yang Liu, Fereshteh Jafari, Haiyang Wang

**Affiliations:** 1 Biotechnology Research Institute, Chinese Academy of Agricultural Sciences, Beijing, 100081 China; 2 State Key Laboratory for Conservation and Utilization of Subtropical Agro-Bioresources, South China Agricultural University, Guangzhou, 510642 China; 3 Guangdong Laboratory for Lingnan Modern Agriculture, Guangzhou, 510642 China

**Keywords:** Arabidopsis, Shade avoidance syndrome (SAS), Light, Hormones, Signaling

## Abstract

As sessile organisms, plants are unable to move or escape from their neighboring competitors under high-density planting conditions. Instead, they have evolved the ability to sense changes in light quantity and quality (such as a reduction in photoactive radiation and drop in red/far-red light ratios) and evoke a suite of adaptative responses (such as stem elongation, reduced branching, hyponastic leaf orientation, early flowering and accelerated senescence) collectively termed shade avoidance syndrome (SAS). Over the past few decades, much progress has been made in identifying the various photoreceptor systems and light signaling components implicated in regulating SAS, and in elucidating the underlying molecular mechanisms, based on extensive molecular genetic studies with the model dicotyledonous plant *Arabidopsis thaliana*. Moreover, an emerging synthesis of the field is that light signaling integrates with the signaling pathways of various phytohormones to coordinately regulate different aspects of SAS. In this review, we present a brief summary of the various cross-talks between light and hormone signaling in regulating SAS. We also present a perspective of manipulating SAS to tailor crop architecture for breeding high-density tolerant crop cultivars.

## Introduction

For higher plants, light is arguably one of the most important environmental factors that not only provides energy for photosynthesis but also serves as an informational signal to direct their growth and developmental patterns throughout their life cycle, ranging from seed germination to seedling de-etiolation, vegetative growth, reproductive transition, and seed setting (Lau and Deng 2010). Plants have also evolved an internal time-keeping mechanism, the circadian clock, to help plants to anticipate and synchronize various developmental and physiological activities with the daily diurnal light/dark cycle generated by the rotation of the earth around the sun, thus increasing plant fitness (Dodd et al. 2005). The plasticity of plants to environmental light changes is inherited in the sophisticated photosensory systems, which through signal transduction and integration, can massively reprogram the transcriptomic activities, leading to adaptive changes in the developmental programs and physiology.

**Fig. 1:**
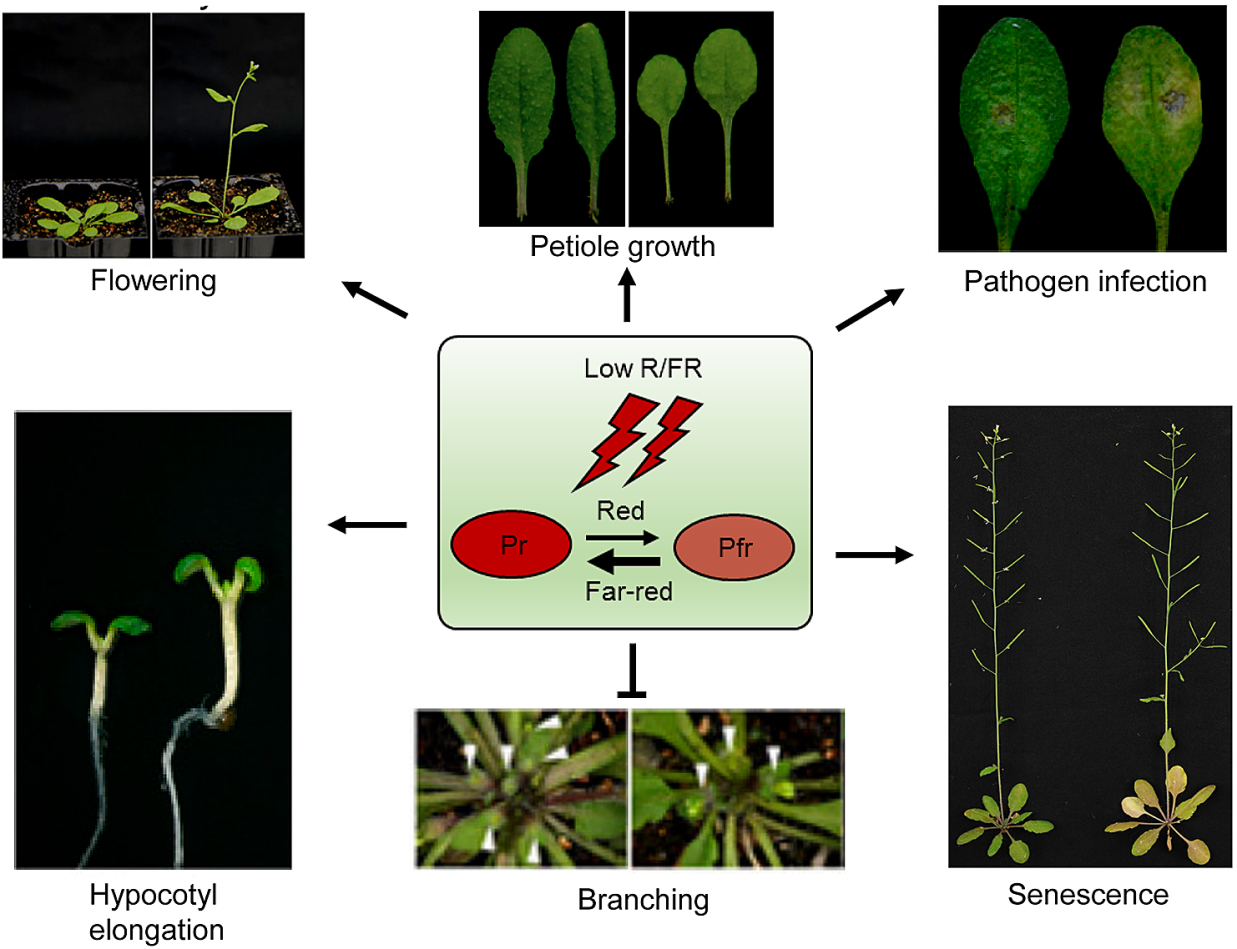
Overview of shade avoidance syndrome. Low R/FR light quality caused by the proximity of competitors induces shade avoidance syndrome. Multiple physiological events, such as accelerated elongation of hypocotyl and petiole, reduced branching, early flowering, precocious senescence and attenuated defense response, are triggered by shade. The wild-type Arabidopsis plant shown on the left is grown under normal white light conditions, while the plants shown on the right is grown under low R/FR ratio conditions. Arrows indicate positive regulation, while bars indicate negative regulation. White arrowheads indicate the short rosette branches. Pictures are adopted from Xie et al. 2017; Liu et al. 2019; Xie et al. 2020a, and 2020b.

Probably a best manifestation of this plasticity is the shade avoidance response, which is triggered when plants sense competition for light from their neighbors. Under a vegetative canopy, the red (R, 600–700 nm) and blue (B, 400–500 nm) spectrum of light is harvested for photosynthesis by upper leaves, whereas far-red (FR, 700–800 nm) light is transmitted or reflected, resulting in a reduction of photoactive radiation (PAR) and reduced R/FR ratios. Plants use a battery of photoreceptors to sense such changes in light quality and quantity, and evoke a suite of adaptive responses collectively termed shade avoidance syndrome (SAS), including stem elongation, reduced branching, hyponastic leaf orientation, early flowering and accelerated senescence (Fig. 1) (Casal 2013; Franklin 2008). These responses are believed to improve individual plant performance and fitness in a crowded plant population, allowing them to finish the life cycle before the canopy getting too dense (Schmitt^1997^). In addition, shade also redirects more carbon resources to growth at the expense of defense, rendering plants more susceptible to pathogen attack (Ballaré 2014). As SAS is a major limiting factor for high-density planting in agricultural practice, it is expected that a better understanding of the molecular mechanisms governing SAS should provide meaningful approaches and target genes for breeding shade-tolerant crop cultivars through manipulating SAS.

## Light regulation of sas in arabidopsis

### Photoreceptors that mediate SAS

Plants use a set of photoreceptor proteins to monitor the changes in light quantity and quality, including phytochromes (phys), cryptochromes (crys), and UV RESISTANCE LOCUS 8 (UVR8). Under shade conditions, changes in R/FR ratios are perceived by phytochromes which exist in two photoreversible forms: the inactive red light-absorbing (Pr) form and the active far-red light-absorbing (Pfr) form. Upon R light absorption, the Pr form is converted to the Pfr form and translocate into the nucleus to activate light-responsive gene expression (Wang and Wang 2015). The Pfr form of phytochromes can switch back to the Pr form upon FR irradiation or through dark reversion (Whitelam et al. 1998). In the model species *Arabidopsis thaliana*, there are five phytochrome receptors (phyA-phyE). Of these, phyB is the predominant phytochrome that suppresses shade avoidance, as the mutant deficient in phyB displays a constitutive shade avoidance phenotype (Franklin 2008). In addition, phyD and phyE play additional roles in suppressing shade avoidance in response to reduced R/FR ratios (Franklin et al. 2003). Interestingly, it has been shown that phyA can antagonize SAS induced by phyB inactivation under deep canopy conditions, thus preventing excess SAS (Martínez-García et al. 2014).

In Arabidopsis, there are two cryptochromes, CRY1 and CRY2, that predominantly regulate blue/UV-A-light mediated photomorphogenesis and flowering time, respectively (Ahmad and Cashmore 1993; Guo et al. 1998). They are photolyase-like proteins existing as physiologically inactive monomers in the dark; blue light irradiation leads to homo-oligomerization of CRYs and altered interactions with various cryptochrome-interacting proteins to transduce the blue light signal (Wang and Lin 2020). Under shade conditions, the drop in B light attenuates CRYs-mediated signaling process and evokes SAS through modulating hormone actions (Keller et al. 2011; Keuskamp et al. 2011; Pedmale et al. 2016). UVR8 acts as the photoreceptor for ultraviolet-B (UV-B, 280–315 nm) and it exists in an inactive dimeric form in cytoplasm. In response to UV-B exposure, it rapidly monomerizes and translocates into the nucleus to initiate the signal transduction process (Kaiserli and Jenkins 2007; Rizzini et al. 2011). Recent studies showed that repression of shade avoidance by UV-B is UVR8-dependent (Hayes et al. 2014).

### Light signaling components that mediate SAS

**Fig. 2:**
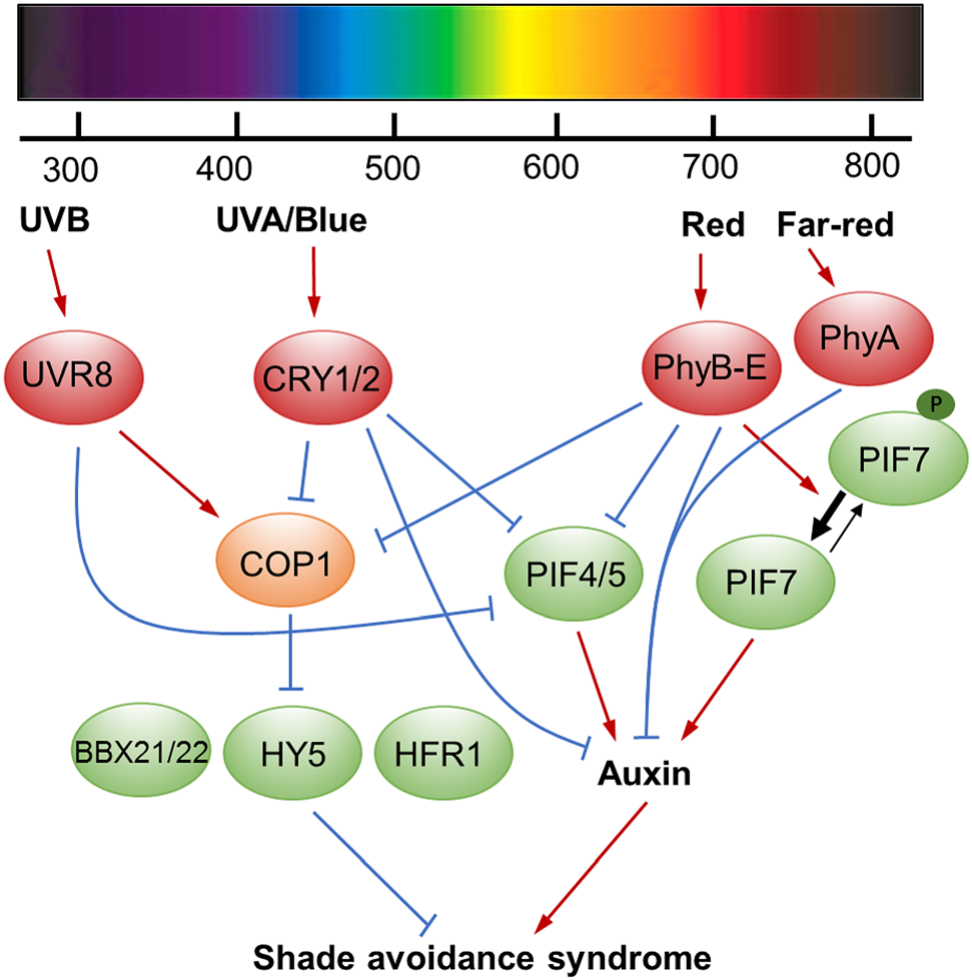
Perception of shade light by photoreceptor signaling network. Photoreceptors detect different light wavelengths and transduce the signal to downstream signaling factors. Low R/FR or low blue light causes inactivation of phytochromes (mainly phyB) or CRYs, respectively, leading to accumulation of PIF4, PIF5 and dephosphorylated PIF7 proteins and activation of auxin-related genes, and thus shade avoidance syndrome. UV-B also represses SAS also through reducing PIF4/5 activity. The light signaling repressor COP1 promotes SAS through degradation of SAS repressors, such as BBX21/22, HY5 and HFR1. Red arrows indicate activation, blue bars indicate inhibition

The mechanisms of phyB-mediated suppression of hypocotyl/stem elongation have been well elucidated now. It has been shown that active phyB are translocated into the nucleus, where they act to represses SAS via inhibiting the activities of a group of positive regulators of SAS, named PHYTOCHROME INTERACTING FACTORS (PIFs), including PIF3, PIF4 and PIF5 and PIF7 (Lorrain et al. 2008; Xie et al. 2017). The association of active phyB with PIFs triggers the phosphorylation of PIF3, PIF4 and PIF5, leading to their proteasome-mediated degradation (Al-Sady et al. 2006; Shen et al. 2007). Under shade conditions, phyB inactivation by low R/FR light stabilizes PIF3/4/5 and allows them to bind and activate downstream targets, mostly auxin biosynthetic genes and cell wall-associated genes involved in promoting stem elongation (Fig. 2). For PIF7, shade promotes nuclear accumulation of the de-phosphorylated form of PIF7, which acts to activate downstream auxin biosynthetic genes to promote SAS (Huang et al. 2018; Leivar et al. 2008; Li et al. 2012).

Moreover, PIF activity is also regulated by a group of SAS negative regulators, such as LONG HYPOCOTYL IN FAR RED 1 (HFR1), PHYTOCHROME RAPIDLY REGULATED1 (PAR1) and PAR2 (Bou-torrent et al. 2011; Buti et al. 2020; Hao et al. 2012). HFR1 and PARs are atypical HLH proteins that do not directly bind DNA, instead they interact with the DNA-binding domain of PIFs, thereby repressing the transcriptional activities of PIFs (Galstyan et al. 2011; Hornitschek et al. 2009). Interestingly, these negative regulators of SAS are rapidly promoted by low R/FR and transcriptionally activated by PIFs, suggesting that these negative regulators of PIFs could serve as a brake mechanism to fine-tune SAS. Additional PIF direct targets include the homeodomain–leucine zipper transcription factor ARABIDOPSIS THALIANA HOMEOBOX PROTEIN2 (ATHB2) and PIF-LIKE1 (PIL1), which are also promoted by low R/FR to fine-tune shade avoidance responses (Hornitschek et al. 2012; Kunihiro et al. 2011). Recently, *FAR-RED ELONGATED HYPOCOTYL3* (*FHY3*) and *FAR-RED IMPAIRED RESPONSE1* (*FAR1*), which encode a pair of transposase-derived transcription factors essential for phyA-mediated FR light signaling in Arabidopsis (Lin et al. 2007), were shown to interact with PIF3/5, and act as negative regulators of SAS as well (Liu et al. 2019, 2020). FHY3 and FAR1 can also directly activate the expression of *PAR1* and *PAR2* to downregulate SAS (Liu et al. 2019).

In addition, several studies have also implicated CONSTITUTIVE PHOTOMORPHOGENIC1 (COP1), ELONGATED HYPOCOTYL5 (HY5) and members of the B-box (BBX) family proteins in SAS regulation. *COP1* encodes an E3 ubiquitin ligase that is accumulated in the nucleus in darkness, where it acts as a central repressor of photomorphogenesis through targeted degradation of transcription activators such as HY5, HFR1 and LONG AFTER FAR-RED LIGHT1 (LAF1). Light triggers the migration of COP1 into the cytosol, thus abolishing its repressive effect on photomorphogenesis (Osterlund et al. 2000; Seo et al. 2003; Yang et al. 2005). Typical SAS requires *COP1*, as SAS and SAS-associated gene expression is suppressed in *cop1* mutants (Roig-Villanova et al. 2006). Interestingly, it was shown that natural or simulated shade conditions can induce rapid nuclear re-accumulation of COP1, and that afternoon shade is more effective than morning shade in inducing nuclear re-accumulation of COP1, implicating a possible role of COP1 in fine-tuning SAS in response to the fluctuating light environments (Pacín et al. 2013). Recently, COP1 was shown to promote the stabilization of PIF3 and PIF5 through repressing BIN2-mediated phosphorylation and degradation (Ling et al. 2017; Pham et al. 2018). Although HY5 does not appear to be involved in the control of hypocotyl growth in response to shade, it was shown to play a critical role in suppressing sunfleck-mediated shade-avoidance response (Roig-Villanova et al. 2006; Sellaro et al. 2011). Notably, the B-box Domain Protein 21 (BBX21) and BBX22 were shown to negatively regulate SAS, whereas BBX24 and BBX25 were shown to promote shade avoidance response (Crocco et al. 2010, 2015), although these BBX proteins are all targeted for 26S proteasome-mediated degradation in a COP1-dependent manner (Chang et al. 2011; Gangappa et al. 2013; Indorf et al. 2007; Xu et al. 2016). The detailed molecular mechanisms underlying the differential roles of these BBX proteins and their regulation await further studies.

Interestingly, recent studies showed that both low blue light (LBL)- and UV-B mediated SAS also acts through modulating PIF4 and PIF5 activity (Fig. 2). It was shown that PIF4 and PIF5 are also cryptochrome-interacting proteins and that PIF4, PIF5, and CRY2 bind to common chromatin regions of target genes (Pedmale et al. 2016). In addition, a role of phyB in suppressing LBL-mediated SAS has also been observed (Pedmale et al. 2016). Moreover, LBL can potentiate low R/FR-induced SAS by increasing PIF5 abundance and attenuating low R/FR-induced gene expression of negative regulators (such as *HFR1*) (de Wit et al. 2016). In addition, it has been shown that B light-activated CRY1 and CRY2 associate with the COP1/SPA1 complex and suppress their ubiquitin ligase activity, thus inhibiting hypocotyl elongation (Liu et al. 2011). Thus, cryptochrome signaling and phytochrome signaling are integrated to modulate plants’ response to a changing environment.

Similarly, it has been shown that UVR8-mediated suppression of hypocotyl elongation also requires degradation of PIF4 and PIF5 (Tavridou et al. 2020a). On the other hand, HFR1 is stabilized under UV-B in a UVR8-dependent manner, which functions in part redundantly with PIL1 to suppress shade-induced gene expression (Tavridou et al. 2020b). In addition, it was recently shown that UV-B induced nuclear accumulation of UVR8 monomeric protein is dependent on COP1 (Yin et al. 2016) and that COP1 interacts with PIF5 to stabilize PIF5 in light-grown plants. Exposure to UV-B promotes the association of UVR8 with COP1, thus disrupting the stabilization of PIF5 by COP1, leads to rapid degradation of PIF5 via the ubiquitin–proteasome system and suppression of SAS (Sharma et al. 2019).

## Integration of light with various hormones in regulating different aspects of sas

It is worth noting that our mechanistic understanding of the signaling pathways of SAS is mostly derived from studies focused on the elongation process in Arabidopsis. Actually, high-density planting elicits diverse responses beyond stem and petiole elongation, including reduced branching, acceleration of flowering time, attenuated defense response and early senescence. These SAS-related physiological responses are regulated by the combined action of light together with a number of plant hormones, including gibberellin (GAs), auxin, brassinosteroids (BR), jasmonic acid (JA), strigolactone (SL), abscisic acid (ABA) and ethylene. Here we describe recent advances on the regulatory mechanisms of various shade avoidance responses by the integration of light with various hormone signaling pathways.

### Light signaling cross-talks with Auxin/BR/GAs signaling to regulate stem elongation

**Fig. 3:**
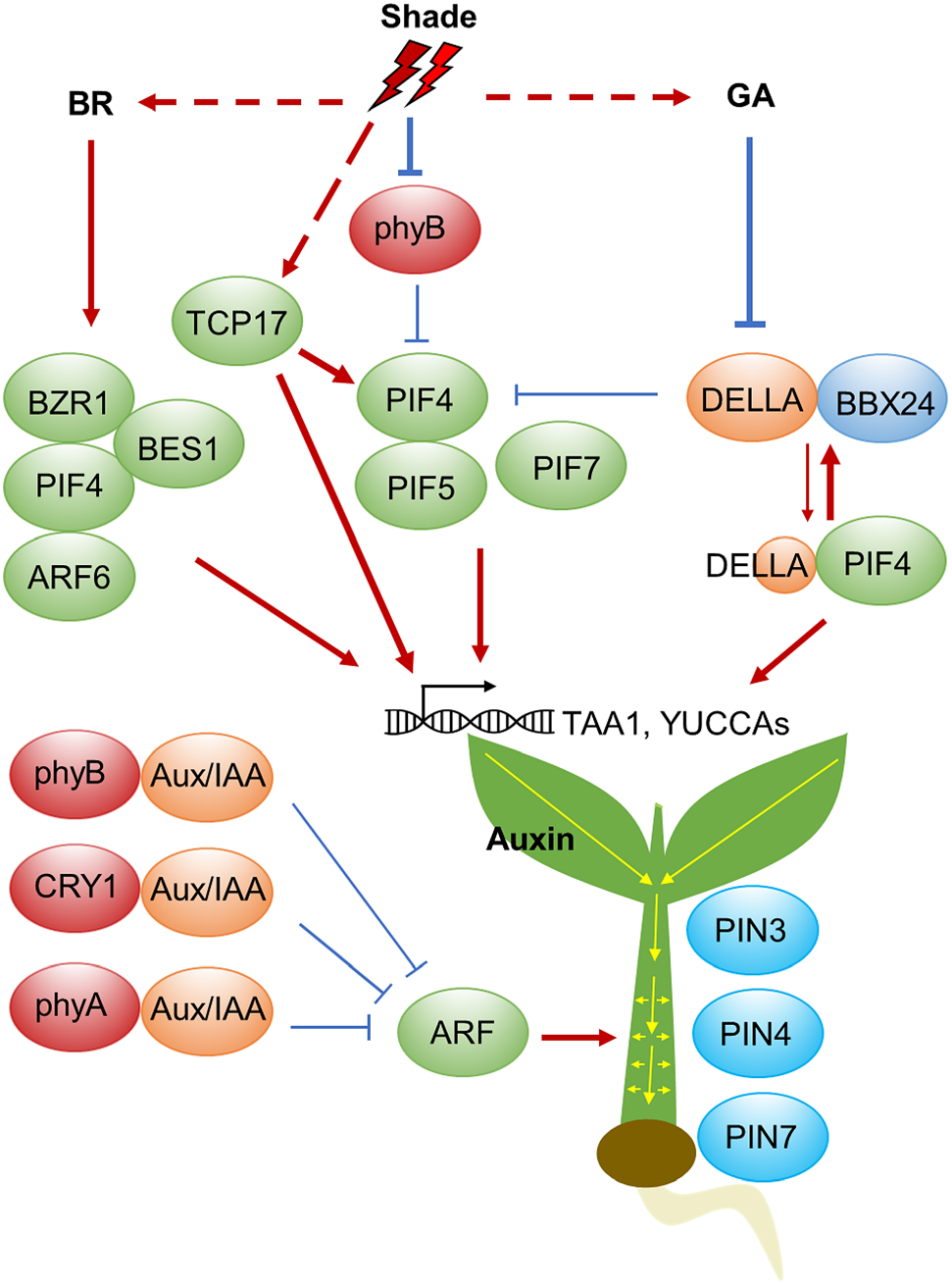
Integration of auxin, BR and GA in shade-mediated stem elongation. Upon shade light, transcription factors PIF4, PIF5 and PIF7 are activated, and GA and BR levels are increased. As a result, BZR1 and BES1 are activated and form a complex with PIF4 and ARF6. DELLA is inhibited through GA-mediated degradation, thus PIF4 is released from repression by DELLA. The transcription regulator BBX24 physically interacts with DELLA and prevents it from interacting with and repressing PIF4. Consequently, auxin biosynthetic genes *TAA1* and *YUCCA* are transcriptionally activated. Low R/FR induces synthesis of auxin in the cotyledons, which is subsequently transported to the hypocotyl via auxin efflux-associated protein PIN-FORMED 3 (PIN3), 4 and 7 to promote growth. Due to direct interaction between photoreceptors and several Aux/IAA proteins, auxin signaling is enhanced as auxin response factors (ARFs) are relieved from Aux/IAA repression. Arrows indicate positive regulation, while bars indicate negative regulation. Bold arrows and bars indicate the events favoured under shade conditions

Auxin plays a major role in shade-induced elongation of hypocotyl, stem and petiole. Under low R/FR conditions, PIF4 and PIF5 are stabilized, while PIF7 is dephosphorylated, and they act together to regulate auxin activity at levels of biosynthesis, transport and signaling (Hornitschek et al. 2012; Iglesias et al. 2018; Li et al. 2012; Sun et al. 2012) (Fig. 3). Consistently, the *pif4 pif5* mutant and mutants of reduced auxin levels, such as higher order *yuc* mutants and *sav3/wei8/taa1*, are defective in low R/FR-induced hypocotyl elongation and other shade avoidance responses (Nozue et al. 2015; Hornitschek et al. 2012). Typically, low R/FR conditions induce auxin synthesis in the cotyledons, which is subsequently transported to the hypocotyl via the auxin efflux-associated protein PIN-FORMED 3 (PIN3), PIN4 and PIN7 to promote hypocotyl growth (Keuskamp et al. 2010; Kohnen et al. 2016; Procko et al. 2014). In particular, shade induces changes in the cellular location of PIN3, which leads to increased free auxin levels in the hypocotyl epidermal cells (Procko et al. 2016). Auxin sensitivity and responsiveness are also enhanced by low R/FR. It has been reported that photoactivated phyB and CRY1 are able to interact with AUX/IAA proteins, and inhibit the binding of AUX/IAAs with the auxin receptor TIR1, thus protecting AUX/IAAs from auxin-induced degradation, resulting in impaired auxin signaling in high R/FR (Xu et al. 2018). Thus, inactivation of phyB and CRY1 by low R/FR and LBL, respectively, could lead to enhanced auxin signaling and SAS. Moreover, it was recently shown that photoactivated CRY1 and phyB can physically interact with ARF6 and ARF8, and repress their DNA-binding activity on downstream target genes, thus inhibiting auxin-induced hypocotyl elongation (Mao et al. 2020). A recent study also showed that a member of TEOSINTE BRANCHED1, CYCLOIDEA, and PCF (TCP) family, TCP17, promotes shade avoidance through upregulating *PIF4* level and auxin biosynthesis (Zhou et al. 2018).

While the above studies primarily focused on the early events of SAS, a recent study provided novel insight into SAS under persistent shade when auxin levels have declined to the prestimulation values. It was found that the sustained inactivation of phytochrome B under persistent shade leads to altered *PIF4* expression profiling, thus modifying auxin perception and signaling to sustain SAS without enhancing auxin levels (Pucciariello et al. 2018).

GAs is another hormone that promotes stem and petiole growth. Low R/FR condition increases GA level partly through transcriptional upregulation of the GA synthesis genes *GA20ox1* and *GA20ox2* (Hisamatsu et al. 2005). DELLA proteins are a subset of plant-specific GRAS family regulators that repress GA signaling, and they are degraded by the SCF^SLY1/GID2^ complex in a GA-dependent manner (Davière and Achard 2013). Interestingly, it was shown that DELLA is able to interact with PIFs and suppress their activities (De Lucas et al. 2008; Feng et al. 2008). Low R/FR ratios or phyB inactivation promote DELLA degradation, resulting in relief of PIFs to promote hypocotyl/stem growth (Djakovic-Petrovic et al. 2007; Leone et al. 2014). Moreover, it was shown that the transcriptional regulators BBX24 and BBX25 physically interact with the DELLA protein GAI and prevent it from interacting with and repressing PIF4, thus promoting GA-induced cell elongation (Crocco et al. 2015). In addition, COP1 can directly regulate DELLA protein stability, because DELLA is targeted for degradation by COP1 in response to shade signal (Blanco-Touriñán et al. 2020).

Shade also stimulates stem growth through coordinating brassinosteroids signaling. The BR signaling components BR-ENHANCED EXPRESSION (BEE) and BES1-INTERACTING MYC-LIKE (BIM) are positive regulators of SAS (Bou-torrent et al. 2013). Importantly, the central BR signaling regulator BRASSINAZOLE RESISTANT1 (BZR1), together with ARF6 and PIF4, form a regulatory module known as the BAP module, which is activated to coordinate growth in response to multiple growth-regulating signals, such as shade (Bouré et al. 2019; Oh et al. 2014). This BAP module may also include BRI1 EMS SUPPRESSOR1 (BES1), which can interact with PIF4, and thus switching BES1 from a repressor to an activator (Martínez et al. 2018). A recent study showed that PIF4, PIF5 and PIF7 act redundantly to upregulate *BR SIGNALING KINASE5* (*BSK5*) expression in shade conditions, leading to activation of the BES1/PIF4/PIF5 signaling module (Hayes et al. 2019). In addition, BR and GA also act interdependently to promote hypocotyl growth, as the DNA-binding activity of BZR1 is inhibited by DELLA (Bai et al. 2012). Therefore, shade activates auxin, GA and BR signaling pathways to synergistically activate downstream central transcription factors, such as the BAP module, to promote stem growth and other shade avoidance responses.

PHYA is known to play a major role in suppressing SAS under deep shade (Martínez-García et al. 2014), however, the underlying molecular mechanism has remained obscure. Yang et al. (2018) recently showed that the accumulation of PHYA is increased under shade, which releases the repressive auxin/indole-3-acetic acid (AUX/IAA) proteins from SCF^TIR1^-mediated degradation, thus weakening auxin signaling and negatively regulating shade responses. Moreover, it was recently shown that under deep canopy, nulcear accumulation of PHYA is promoted, causing reduced COP1 nuclear speckles and subsequent changes in downstream target genes (PIF4, PIF5 and HY5), and consequently inhibits SAS through modulating BES1/BZR1 and BR signaling (Song et al. 2020).

It is also worth mentioning that reduced R/FR ratios can promote phototropism of de-etiolated seedlings (reorientation of hypocotyl growth) through phyB-controlled auxin biosynthesis, to avoid canopy shade (Goyal et al. 2016). A recent study further showed that persistent LBL promotes seedling phototropism and that this response is also regulated by phyB and the CRY1-PIF4 module through modulating auxin signaling in the hypocotyls, thus reinforcing a critical role of the CRY1-PIF4 module in regulating different light-mediated responses (Boccaccini et al. 2020).

### Light and the miR156/SPL module control SL- and ABA-mediated regulation of branching

Branching (tillering in cereal crops) is a major component of plant architecture and a critical determinant of crop productivity. Shade suppresses axillary bud outgrowth and thus reducing branching. phyB inactivation or *PIF4/PIF5* overexpression leads to branching repression in both high and low R/FR light conditions (Holalu et al. 2020; Reddy and Finlayson 2014; Xie et al. 2017). A major genetic pathway regulating branch outgrowth is the *TB1/FC1/BRC1* pathway that represses axillary bud outgrowth in both monocots and dicots (Wang et al. 2019). Recent studies showed that under canopy shade conditions, inactivation of phyB causes elevated *BRC1* expression in the axillary buds in a manner dependent on PIF4 and PIF5 (Finlayson et al. 2010; Holalu et al. 2020).

**Fig. 4:**
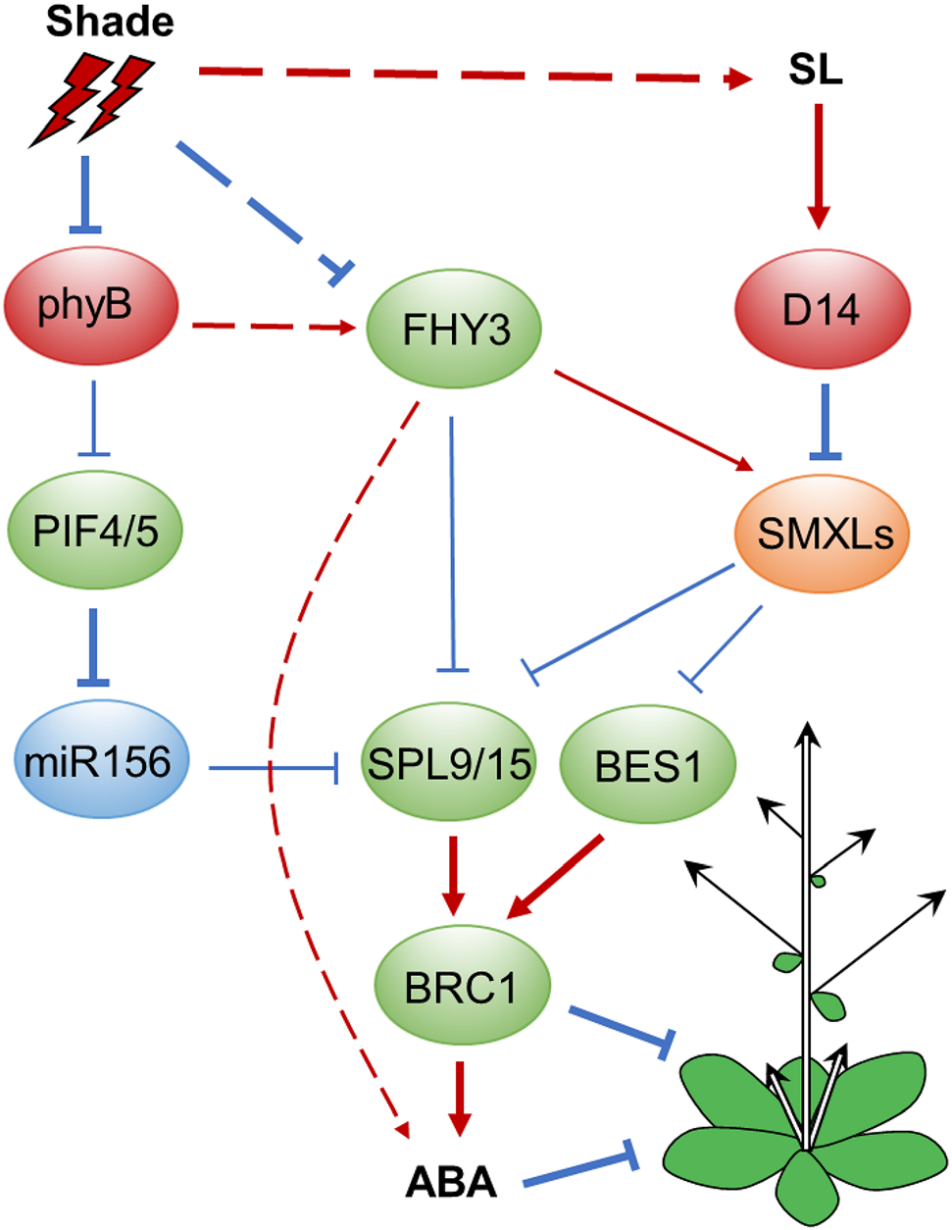
The crosstalk between SL and ABA in shade-mediated shoot branching. Shade-mediated PIF4/5 activation represses *MIR156* expression, thereby releasing its targets SPL9/15 to promote *BRC1* expression and repress branching. Shade conditions also downregulate FHY3 protein accumulation, thus reliefing SPL9 and SPL15 from inhibition by FHY3, leads to activation of *BRC1* expression. The SL repressor proteins SMXLs are directly up-regulated by FHY3. SMXL proteins can interact with SPL9/15 and BES1, and repress their transcriptional activity on *BRC1*, thereby regulating branching. ABA acts downstream of *BRC1* and *FHY3* to regulate branching. Arrows indicate positive regulation, while bars indicate negative regulation. The dotted lines indicate either indirect regulation or regulation in an unknown manner. Bold arrows and bars indicate the events favoured under shade conditions

Several studies have shown that the miR156-SQUAMOSA-PROMOTER BINDING PROTEIN-LIKE (SPL) module plays an important role in controlling *FC1*/*BRC1* expression (Jiao et al. 2010; Wang et al. 2015; Xie et al. 2020a). It has been shown that Arabidopsis SPL9/15 and rice OsSPL14 are able to directly bind to the *BRC1* and *FC1* promoter, respectively, and activate their transcription (Song et al. 2017; Xie et al.2020a). Mechanistically, inactivation of phytochrome B under shade conditions promotes accumulation of PIFs, which directly bind to the promoters of multiple *MIR156* genes and repress their expression, resulting in the release of downstream *SPL* genes to promote SAS (Xie et al. 2017). The upregulation of *BRC1* by SPL proteins contributes to reducing branching (Fig. 4). Moreover, it was shown that FHY3 and FAR1 interact with both SPL9 and SPL15 and inhibit their binding to the *BRC1* promoter, and that simulated shade conditions downregulate the accumulation of FHY3 and FAR1 proteins, thereby upregulating *BRC1* level and suppressing branching (Xie et al. 2020a). Together, these findings suggest that two sets of SAS regulators, FHY3/FAR1 and PIFs, control branching through oppositely modulating SPL activity/expression and thus *BRC1* expression.

It is known that tiller bud growth is also repressed by a carotenoid-derived hormone strigolactone. SL represses bud outgrowth and branching through promoting the degradation of a set of central repressor proteins, DWARF 53 (D53) in rice and D53-like SUPPRESSOR OF MORE AXILLARY GROWTH2-LIKE proteins (SMXL6/7/8) in Arabidopsis, which relieve their transcriptional inhibitory effect on SPL-activated *BRC1*/*FC1* expression (Song et al. 2017; Wang et al. 2018). Strikingly, at least two *SMXLs* (*SMXL6* and *SMXL7*) are directly activated by FHY3 and FAR1. Moreover, SMXL6, SMXL7, and SMXL8 can physically interact with SPL9/15 and inhibit their transcriptional activation activities on *BRC1* (Xie et al., 2020a). Additionally, SMXLs can also interact with BES1 and repress its transcriptional activation activity on *BRC1* to regulate branching (Hu et al. 2020). Taken together, shade decreases the protein abundance of FHY3/FAR1 and attenuates the expression of *SMXL6/7*, relieving SPL9/15 and BES1 proteins to activate *BRC1*, thus repressing branching (Fig. 4).

Shade-induced suppression of branching also involves the action of the phytohormone ABA (Fig. 4). Under shade conditions, ABA concentration and signaling particularly in lower buds are increased partly through PIF4/5-mediated upregulation of some ABA biosynthetic and responsive genes. Consistently, ABA synthesis mutants exhibit increased branching and lack of responsiveness to low R/FR (Holalu et al. 2020; Reddy et al. 2013). However, ABA-mediated inhibition of branching might not act through the *BRC1* pathway, as exogenous ABA treatment does not alter *BRC1* level (Yao et al. 2015). Instead, ABA was shown to act downstream of *BRC1* to suppress bud development (González-Grandío et al., 2017). Interestingly, IAA accumulation as well as expression of auxin biosynthetic and transport genes are repressed by ABA treatment, suggesting that auxin is involved in ABA-mediated repression of bud outgrowth. Additionally, it has been shown that *FHY3* and *FAR1* are positive regulators of ABA signaling, as ABA sensitivity as well as expression of ABA responsive genes are attenuated in the *fhy3* and *far1* mutants (Tang et al. 2013). Thus, it will be interesting to explore the connection between FHY3/FAR1 and ABA-mediated branching suppression.

### The action of light, miR156/SPL and GA in flowering

Flowering is a major developmental transition in the plant life cycle, and proper flowering time control is essential for plants’ reproductive success and survival (Amasino and Michaels 2010). In response to competition for light from their neighbors, shade-intolerant plants flower precociously to ensure reproductive success and survival. The Arabidopsis wild-type plants subjected to low R/FR-treatment and *phyB* mutants grown under high R/FR demonstrate accelerated flowering (Wollenberg et al. 2008). The underlying mechanism involves promoted expression and protein stability of the core flowering regulator CONSTANS (CO), which acts to induce expression of the florigen gene *FLOWERING LOCUS T* (*FT*) and its close homolog *TWIN SYSTER OF FT* (*TSF*) (Halliday et al. 2003; Valverde et al. 2004; Wollenberg et al. 2008). It was recently shown that PIF4, PIF5 and PIF7 play a predominate role in shade-induced flowering, through directly upregulating *FT*/*TSF* while downregulating *Pri-MIR156E/F* (Zhang et al. 2019; Galvāo et al. 2019). In addition, it was shown that under shade conditions, GA-mediated degradation of DELLA relieves their inhibition on PIF4, which in turn activates *FT* transcription to promote flowering (De Lucas et al. 2008; Kumar et al. 2012; Yamaguchi et al. 2014).

**Fig. 5:**
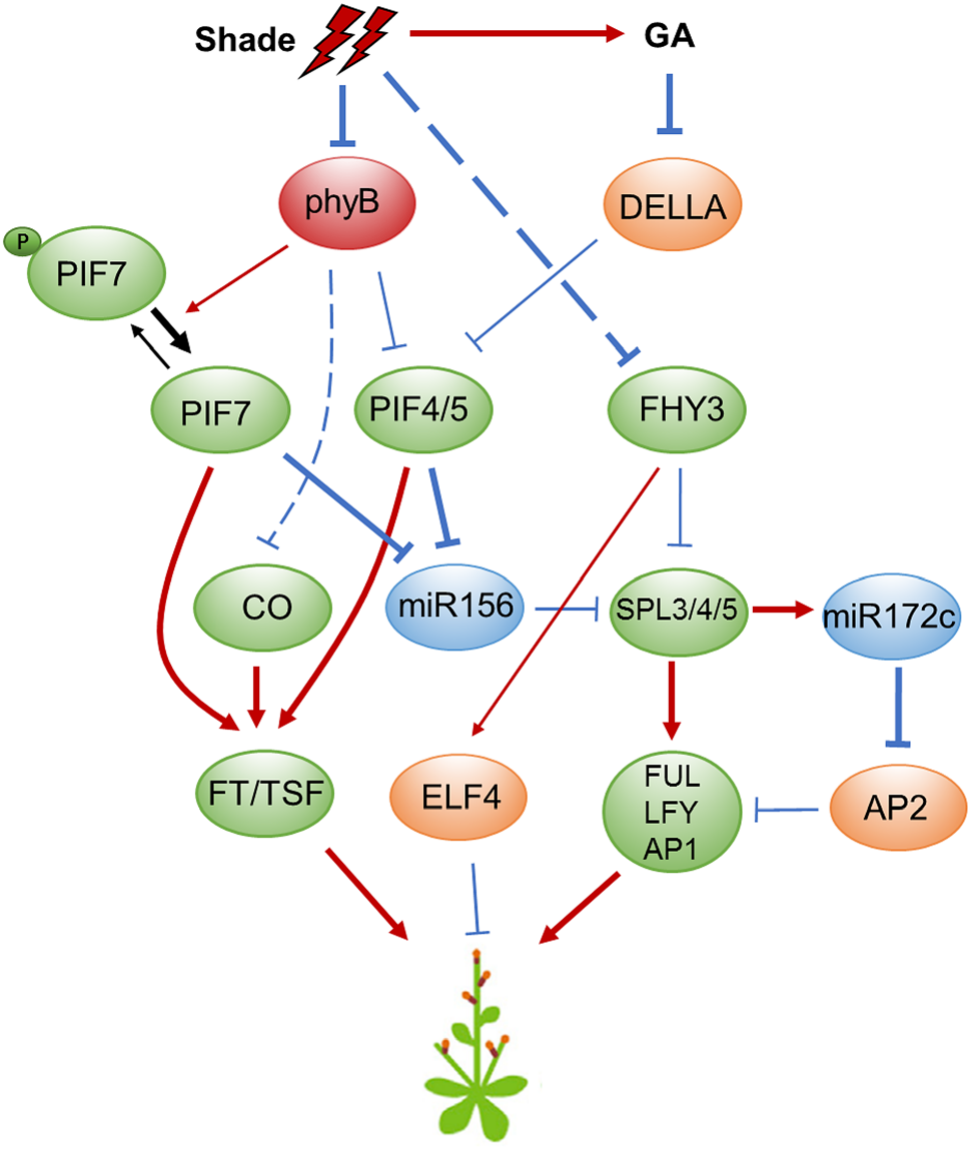
The network of shade-regulated flowering time. Upon shade light, the increased activity and abundance of PIFs and CO lead to upregulation of *FT* or *TSF*, thus accelerating flowering. Additionally, SPL3/4/5 are released from repression by FHY3 and miR156, which in turn promotes flowering through upregulating several flowering promoting factors, such as *FUL*, *LFY*, *AP1* and *miR172*. Arrows indicate positive regulation, while bars indicate negative regulation. The dotted lines indicate either indirect regulation or regulation in an unknown manner. Bold arrows and bars indicate the events favoured under shade conditions

*FHY3* and *FAR1* were shown to negatively regulate flowering time under both long-day and short-day conditions through transcriptional regulation of *Early Flowering4* (*ELF4*) (Li et al. 2011). A recent study revealed that FHY3 and FAR1 can physically interact with three flowering-promoting SPL transcription factors (SPL3, SPL4, SPL5) and inhibit their binding to the promoters of several key flowering regulatory genes, including *FRUITFUL* (*FUL*), *LEAFY* (*LFY*), *APETALA1* (*AP1*), and *MIR172C*, thus downregulating their transcript levels and delaying flowering. Under simulated shade treatments, the levels of transcripts and proteins of FHY3 and FAR1 decrease, and thus more SPL3/4/5 proteins are released from inhibitory interactions with FHY3 protein, resulting in increased expression of *FUL*/*LFY*/*AP1*/*MIR172C* and thus early flowering (Xie et al. 2020b) (Fig. 5).

### The action of JA and SA in shade mediated growth-defense tradeoff

**Fig. 6:**
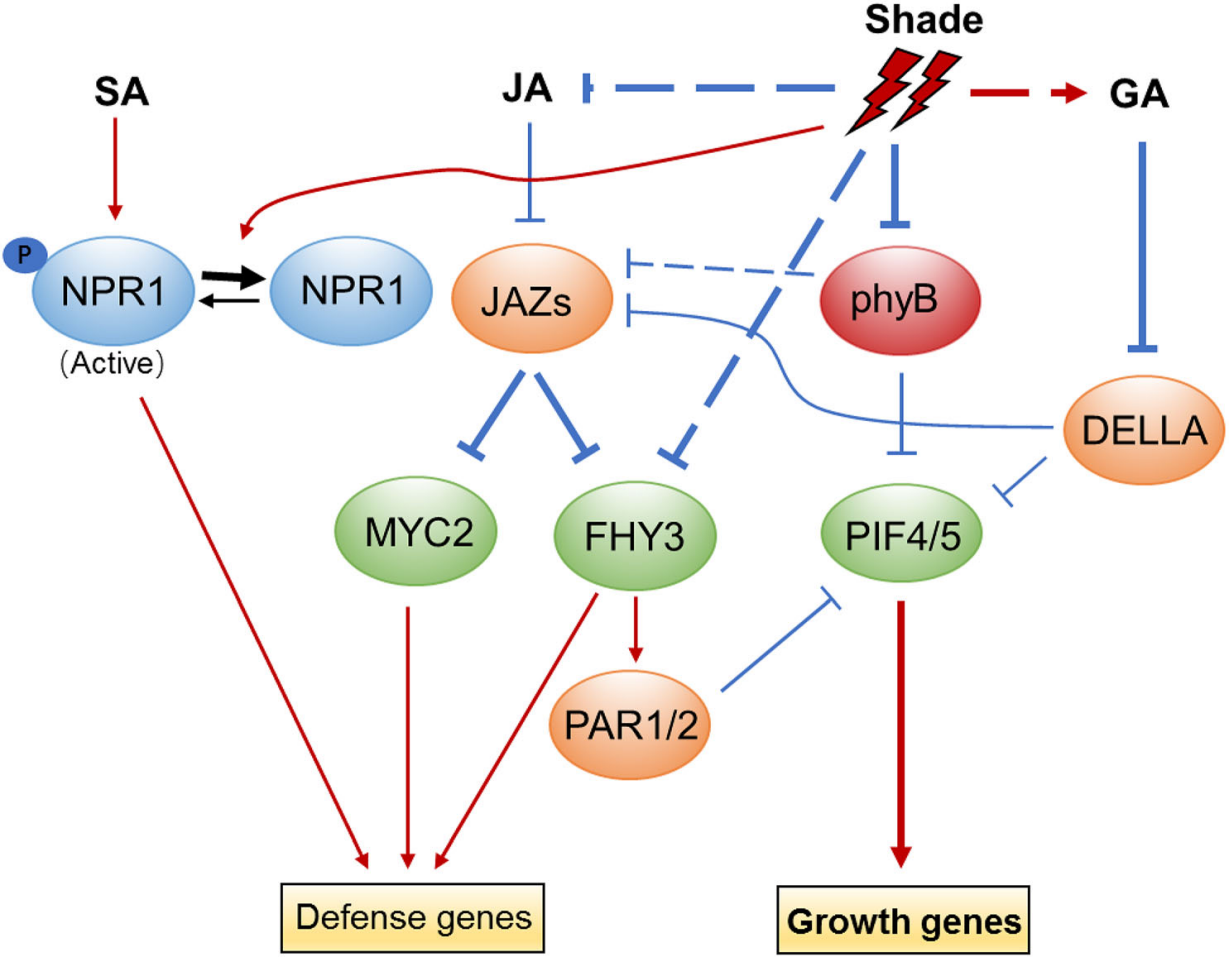
The action of JA and SA in shade-mediated growth and defense tradeoff. The phosphorylated NPR1, which is the active form in SA signaling is reduced under shade conditions. Due to reduced JA level and the repression of phyB and DELLA in shade conditions, JAZ proteins are stabilized and are able to repress the transcriptional activity of MYC2 and FHY3. Therefore, SA- and JA-related targets of defense-responsive gene expression are reduced. Meanwhile, PIF4 and PIF5 are accumulated and released from repression by DELLA and PAR1/2, thus promoting growth-related target genes expression. Arrows indicate positive regulation, while bars indicate negative regulation. The dotted lines indicate either indirect regulation or regulation in an unknown manner. Bold arrows and bars indicate the events favoured under shade conditions

Another important aspect of SAS is attenuated resistance to abiotic and biotic stresses, as limited resources are reallocated from defense toward rapid elongation under shade conditions (de Wit et al. 2013). Resistance against a hemibiotrophic pathogen (*Pseudomonas syringae* pv tomato) and a necrotrophic pathogen (*Botrytis cinerea*) was found to be suppressed by shade treatment (Cerrudo et al. 2012; de Wit et al. 2013; Pieterse 2013). JA is a core regulatory hormone that orchestrates defense response against insects and necrotrophic pathogens (Browse 2009). It is known that low R/FR ratios repress JA-induced defense responses, including JA signaling and the expression of defense-related genes against herbivores and pathogens. Under shade conditions, inactivation of phyB results in attenuated JA sensitivity through promoting the stability of PIFs and JAZ proteins, while destabilizing DELLA proteins, thus relieving PIFs and JAZs from the inhibitory effect of DELLAs and allowing them to activate downstream genes and promote growth at the expense of defense (Cerrudo et al. 2012; Cortés et al. 2016; de Wit et al. 2013; Moreno et al. 2009; Yang et al. 2012) (Fig. 6). Besides PIFs, FHY3 and FAR1 were recently shown to modulate JA-mediated defense responses, as FHY3 and FAR1 are able to interact with both JAZs and MYC2 (Liu et al. 2019). Interestingly, the *fhy3 far1* mutant displayed reduced JA sensitivity and increased susceptibility to necrotrophic fungus *Botrytis cinerea* under both high and low R/FR conditions. Consistently, expression of several typical JA-responsive genes, including *LOX2*, *PDF1.2*, *TAT1*, and *VSP2*, was significantly reduced in the *fhy3 far1* mutant. The transcription activation of JA responsive genes by FHY3 can be repressed by JAZ1 through direct interaction. In parallel, FHY3 and MYC2 form heterodimers and coordinately induce the expression of JA-responsive genes. The dual functions of FHY3 in regulating growth and defense under shade conditions indicate that plants balance their growth and defense responses through the convergence of the phytochrome signaling and JA signaling pathways. On one hand, FHY3 and FAR1 activate the expression of *PAR1*/*PAR2*, which inhibit the expression of growth-related genes by forming non-DNA binding heterodimers with PIFs, thus preventing an exaggerated elongation growth. On the other hand, FHY3/FAR1, together with MYC2, activate the expression of defense-associated genes, while JAZ proteins inhibit the activity of FHY3/FAR1 and MYC2 to maintain a proper level of defense gene expression and defense response (Liu et al. 2019).

In addition to JA, salicylic acid (SA)-dependent disease resistance is also inhibited by reduced R/FR ratios (Fig. 6). Although SA level is not altered under low R/FR light, SA-dependent phosphorylation of NPR1, a key transcriptional regulator of SA-mediated defence, is reduced, thus resulting in inhibition of SA-induced transcription of disease resistance-related genes (de Wit et al. 2013; Nozue et al. 2018).

### Light and ethylene signaling modulates senescence

The volatile hormone ethylene has also been shown to act as a proximity perception signal within canopies. Low R/FR-induced petiole elongation is absent in the ethylene signaling mutants *ein2* and *ein3 eil1*, indicating a requirement of the intact ethylene signaling pathway for shade avoidance response in Arabidopsis (Pierik et al. 2009). In accordance with this, transgenic tobacco lacking sensitivity to ethylene showed delayed shade‐avoidance traits including leaf angles and stem elongation in response to shade signal (Pierik et al. 2003). Presumably, ethylene signaling acts downstream of photoreceptors to regulate SAS (Pierik et al. 2004, 2007). In support of this notion, Shi et al. (2016) showed that light-activated phyB can physically interact with EIN3 and lead to its rapid degradation. Thus, low R/FR conditions may stabilize EIN3 due to inactivation of phyB, and in turn promoting SAS.

**Fig. 7:**
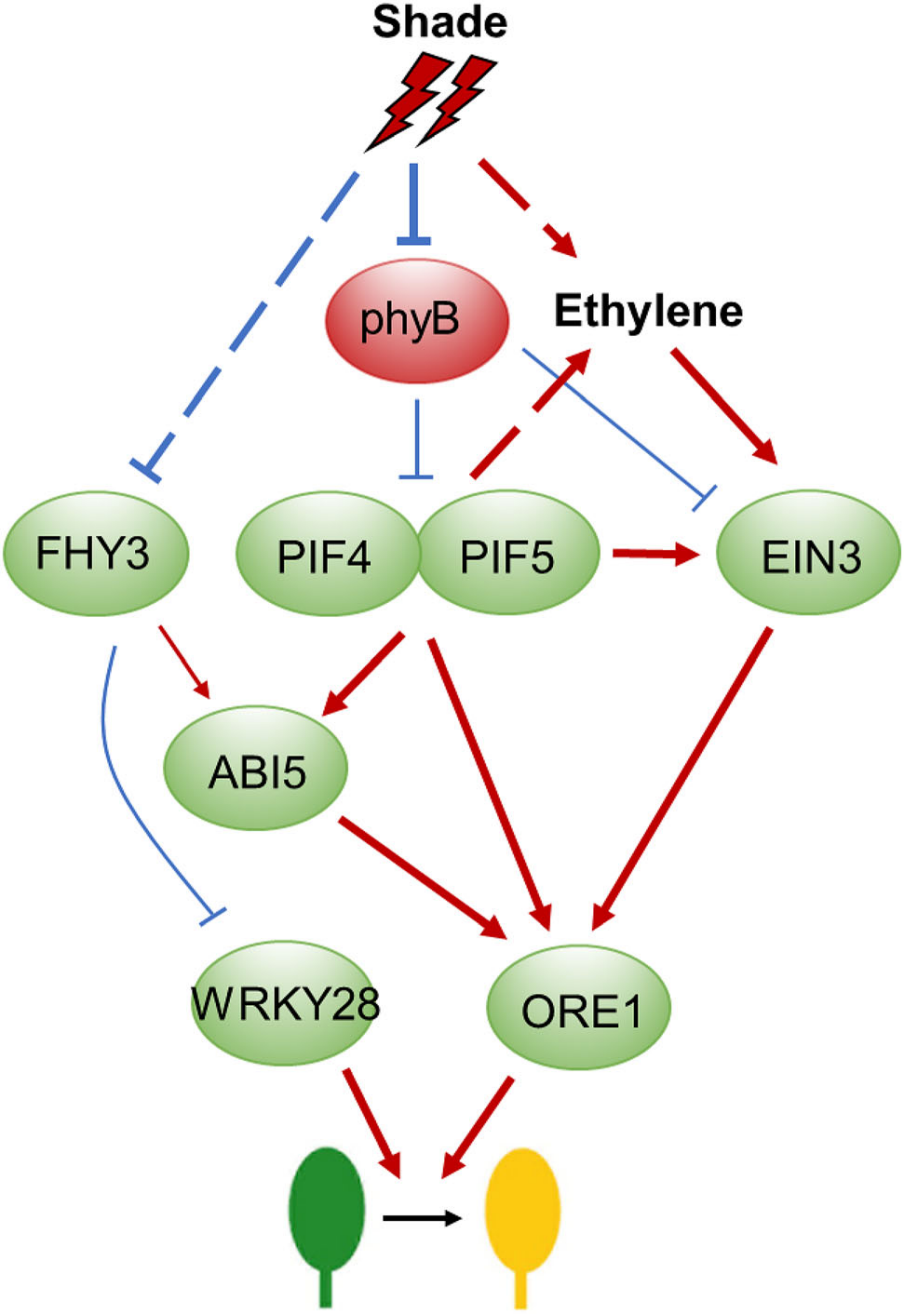
Integration of light with ethylene promotes senescence under shade conditions. Under shade conditions, the activity and abundance PIF4/5 and EIN3 are increased by the action of ethylene or shade light. As a result, the key senescence regulator *ORE1* is up-regulated directly by PIF4/5, EIN3 and ABI5. Meanwhile, WRKY28 is relieved from FHY3 repression. Therefore, the upregulated *ORE1* and *WRKY28* promote senescence under shade light. Arrow indicates positive regulation, while bar indicates negative regulation. The dotted lines indicate either indirect regulation or regulation in an unknown manner. Bold arrows and bars indicate the events favoured under shade conditions

Another important effect of shade on the whole plant is precocious leaf senescence, a process of aging triggered by ethylene (Fig. 7). It was shown that leaf senescence is attributable to accelerated degradation of chlorophylls and proteins under shade conditions (Brouwer et al. 2012). Consistently, it has been shown that PIF4 negatively regulates chloroplast activity through activating expression of the chlorophyll degradation gene *Non-yellowing 1* (*NYE1*) and repressing expression of the chloroplast activity maintainer gene *Golden 2-like Transcription factor 2* (*GLK2*) (Song et al. 2014). In addition, PIF4 and PIF5 are able to promote dark-induced senescence through directly activating ethylene biosynthesis genes and key transcription factors of the ethylene and ABA signaling pathways, such as *ACSs*, *EIN3* and *ABI5*, thereby activating downstream senescence-related targets, such as *ORE1.* Photoactivated phyB can inhibit leaf senescence through repressing leaf senescence activators PIF4/PIF5 at the post-translational level (Sakuraba et al. 2014; Song et al. 2014). A recent report revealed that the *fhy3* and *far1* mutants also exhibit precocious leaf senescence in both high and low R/FR light conditions, indicating a negative role of *FHY3/FAR1* in regulating leaf senescence (Tian et al. 2020). It was shown that one mechanism of FHY3/FAR1 suppressing senescence is through repressing the expression of *WRKY28*, a positive regulator of senescence (Tian et al. 2020). Given the demonstrated interaction between FHY3/FAR1 with PIFs (Liu et al. 2020), it will be interesting to investigate how these two sets of SAS molecules act coordinately to regulate leaf senescence in the future.

## Perspective and conclusions

This review summarizes our present understanding of the integration of light and hormone signaling pathways in the regulation of multiple physiological responses of SAS in Arabidopsis. It is hoped that such knowledge can be leveraged to increase our understanding of SAS in various crops. Due to the global climate change and rising population, improvement of yield production per unit area is becoming increasingly important. Much effort has been devoted to breeding crops with the increased yield at high-planting density. However, SAS in crops can be detrimental to yield, due to increased lodging, reduced biomass production, precocious maturation and reduced defense to pathogens, etc. Therefore, SAS needs to be attenuated for genetic improvement of crops.

Indeed, much effort has been made to attenuate SAS in several crops such as rice, wheat, potato, tomato, and turfgrasses by overexpressing *PHYA* or *PHYB*. In most case, limited success has been achieved due to associated pleiotropic effects (Boccalandro et al. 2003; Boylan and Quail 1989; Ganesan et al. 2012; Garg et al. 2006; Gururani et al. 2015; Robson et al. 1996; Thiele et al. 1999). Thus, a better understanding of SAS in various crops is urgently needed to fine-tune SAS and tailor plant architecture for adapting to high-density planting. Several recent studies have made progress to meet this need. A recent study reported that overexpression of *GmCRY1b* in soybean conferred improved plant architecture (such as reduced plant height, and thus more lodging-resistant) and higher yield performance under density planting or maize-soybean intercropping conditions. Moreover, it was shown that *GmCRY1s* play important roles in mediating LBL-induced SAS through modulating GA metabolism (Lyu et al. 2020). Other studies in maize reported that the *Zmphyb1 Zmphyb2* double mutant displays constitutive shade avoidance responses, while *Zmphyc1 Zmphyc2* double mutant shows moderately early flowering under long-day conditions (Kebrom et al. 2006; Li et al. 2020; Sheehan et al. 2007). Furthermore, expression of these two hyperactive mutant forms of *ZmPHYB1* or overexpression of *ZmPHYCs* effectively reduces the plant height and ear height of mature maize plants in field conditions (Li et al. 2020; Zhao et al. 2020). Additionally, the *Zmpifs* knockout mutants also display attenuated SAS and reduce plant and ear height effectively (Wu et al. 2019). Despite the progress been made, much more work remains to be done to provide a comprehensive understanding of SAS in crops and to identify valuable targets for genetic improvement of SAS. As various hormones are intimately involved in regulating different aspects of SAS, components of their signaling pathways may provide valuable targets for more precise manipulation of the various agronomic traits of crops (such as biomass, plant height, branches, leaf angle, leaf size, senescence, flowering time, etc.). On the other hand, tapping into the rich resource of natural variation of crop germplasm using population genetics approaches may offer an alternative approach to identify the superior alleles associated with attenuated SAS for breeding shade-tolerant crop cultivars (Wang et al. 2020). With the rapid advances in plant functional genomics, genome editing technologies and synthetic biology, more tools and resources will become available to meet the challenge of breeding high-density tolerant crops with higher efficiency.
