# Correction: Integration of light and hormone signaling pathways in the regulation of plant shade avoidance syndrome

**DOI:** 10.1007/s42994-023-00136-2

**Published:** 2024-01-22

**Authors:** Yang Liu, Fereshteh Jafari, Haiyang Wang

**Affiliations:** 1 Biotechnology Research Institute, Chinese Academy of Agricultural Sciences, Beijing, 100081 China; 2 State Key Laboratory for Conservation and Utilization of Subtropical Agro-Bioresources, South China Agricultural University, Guangzhou, 510642 China; 3 Guangdong Laboratory for Lingnan Modern Agriculture, Guangzhou, 510642 China

**Correction: aBIOTECH (2021) 2:131–145** 10.1007/s42994-021-00038-1

In this article the author’s name Fereshteh Jafari was incorrectly written as Feresheeh Jafari.

The original article has been corrected.

